# Effect of diet on pathogen performance in the microbiome

**DOI:** 10.20517/mrr.2021.10

**Published:** 2022-03-26

**Authors:** Ronan Strain, Catherine Stanton, R. Paul Ross

**Affiliations:** ^1^APC Microbiome Ireland, Biosciences Institute, University College Cork, Cork T12 YT20, Ireland.; ^2^Teagasc Food Research Centre, Moorepark, Fermoy, Co. Cork P61 C996, Ireland.; ^3^School of Microbiology, University College Cork, College Road, Cork T12 K8AF, Ireland.

**Keywords:** Colonisation resistance, gut microbiota, pathogen, diet, infection

## Abstract

Intricate interactions among commensal bacteria, dietary substrates and immune responses are central to defining microbiome community composition, which plays a key role in preventing enteric pathogen infection, a dynamic phenomenon referred to as colonisation resistance. However, the impact of diet on sculpting microbiota membership, and ultimately colonisation resistance has been overlooked. Furthermore, pathogens have evolved strategies to evade colonisation resistance and outcompete commensal microbiota by using unique nutrient utilisation pathways, by exploiting microbial metabolites as nutrient sources or by environmental cues to induce virulence gene expression. In this review, we will discuss the interplay between diet, microbiota and their associated metabolites, and how these can contribute to or preclude pathogen survival.

## INTRODUCTION

The gut microbiota has coevolved with its host over millions of years and augments the coding potential of the human genome (~22,000 genes) by upwards of 500-fold^[1]^. Indeed, the human genome itself, encodes at most only 17 enzymes involved in food digestion, mainly the digestion of starch, sucrose and lactose^[2]^. On the other hand, our gut microbiota encodes upwards of 60,000 Carbohydrate-Active Enzymes, with diverse specificities, facilitating the depolymerisation and fermentation of complex dietary polysaccharides into host utilisable short-chain fatty acids (SCFAs)^[3]^. This gut microbiota is heterogeneous and highly personalised, and while bacterial enterotypes cluster independently of nationality, ethnicity, sex, age, or body mass index^[4]^, diet is the dominant selective force that defines microbiota membership and functionality^[5]^. Diet is also the simplest to customise and therefore presents the most straightforward route for therapeutic intervention.

The distribution of bacteria throughout the gastrointestinal (GI) tract varies, from 10^3^-10^4^ cells/mL in the stomach and upper small intestine, to 10^11^ cells/mL in the colon^[6]^. Furthermore, the taxonomic composition of these communities is niche-specific, and largely defined by the nutritional requirements of the residing bacteria. These contentions are supported by observations in gnotobiotic mouse models whereby concentrations of individual dietary components correlate with the relative abundance of specific microbiota members^[7]^. For example, *Bacteroides cellulosilyticus* has the ability to be controlled by administration of different concentrations of the prebiotic fibre arabinoxylan^[8]^. This phenomenon opens up the potential for therapeutic probiotic colonisation, which has been demonstrated by Kearney *et al.*^[9]^; administration of the seaweed polysaccharide polyphyran and a polyphyran-degrading commensal *Bacteroides plebius* enabled successful engraftment of this species.

The majority of human enteric pathogens are part of a small group of bacterial families that belong to the phylum Proteobacteria; the *Enterobacteriaceae*, i.e., *Escherichia coli* (*E. coli*), *Yersinia*, *Salmonella*, *Shigella*; the *Vibrionaceae* (*Vibrio cholera*) and the *Camplyobacteriaceae* (*Camplyobacter*)^[10]^. Bacterial pathogens must overcome an array of obstacles such as oesophageal peristalsis, stomach pH and locating a permissible niche in the intestine in order to access nutrients to begin replication and to achieve successful colonisation in the GI tract. The final hurdle is to overcome resident commensal bacteria of the large intestine. Continuous competition for nutrients, and compartments, the production of antimicrobial substances by commensals, and the barrage of immune responses evoked by the commensals themselves collectively give way to the phenomenon of colonisation resistance. For example, commensal symbionts and their related pathogens often compete with each other for metabolic resources compared with distant unrelated species. These metabolites include diverse carbon sources, bile acids, trace metals and vitamins. An overview of some metabolites influencing pathogen virulence and fitness can be found in Table 1. Those bacteria which possess high-affinity transporters for available nutrients will ultimately define the microbial community structure, but also serve as a barrier for GI pathogens. However, many pathogens confer the ability to subvert competition with commensal bacteria, often by generating their own specific niche to suit their metabolic needs. As an example, invasion of epithelial cells provides an environment suited to intracellular pathogens. Additionally, there is accumulating evidence to suggest that intestinal pathogens/pathobionts may subvert and exploit the host immune response to induce microbial dysbiosis and improve conditions for their subsequent colonisation^[11]^. Furthermore, intestinal pathogens have evolved unique nutrient utilisation pathways in relation to their symbiotic counterparts; *Escherichia* can utilise alternative sugar sources to that of their commensal rivals^[12]^, and gain a competitive advantage. In this regard, what we consume may have the potential to alter bacterial networks and shift the balance in favour of or against pathogen survival.

**Table 1:** Metabolites influencing pathogen virulence and fitness

**Pathogen**	**Gene expression**	**Gene function**	**Metabolite**	**Ref.**
EHEC	LEE-encoded T3SS	Adherence	↑Butyrate	[87]
EHEC	LEE-encoded T3SS	Adherence	↑Ethanolamine	[165]
EHEC	LEE-encoded T3SS	Adherence	↑Succinate	[166]
EHEC	LEE-encoded T3SS	Adherence	↑Oxygen	[167]
EHEC	LEE-encoded T3SS	Adherence	↓D-serine	[168]
EHEC	Adhesions	Adherence	↑Ethanolamine	[169]
EHEC	Adhesions	Adherence	↑Choline	[169]
EHEC	iha	Adherence	↑Butyrate	[74]
EHEC	FliC	Motility	↑Acetate	[73]
EHEC	GvrA/LEE-encoded T3SS	Acid resistance	↑Bicarbonate	[170]
EHEC	Qse	Quorum sensing	↑Ethanolamine	[169]
EHEC	Qse	Quorum sensing	↑Fucose	[171]
EHEC	Stx	Toxin	↑Ethanolamine	[169]
EHEC	SdhA	Respiration	↑Fumarate	[172]
EHEC	EspA/EspB	A/E lesions	↑Indole	[134]
AIEC	tdc/sda	Inflammed gut colonisation	↑L-serine	[137]
Salmonella	SPI-1	Invasion	↑Acetate	[68]
Salmonella	SPI-1	Invasion	↑Formate	[66]
Salmonella	Hyb	Invasion	↑Hydrogen	[173]
Salmonella	SPI-2	Intracellular replication	↑Ethanolamine	[174]
Salmonella	*ttrSR ttrBCA*	Inflammed gut respiration	↑Tetrathionate	[175]
Salmonella	fraBDAE	Carbon/nitrogen utilisation	↑Fructose-asparagine	[176]
Salmonella	pduA-X	Inflammed gut respiration	↑1,2-propandiol	[177]
Salmonella	SPI-1	Invasion	↓Indole	[133]
C. difficile	TcdAB	Toxin	↑Butyrate	[178]
C. difficile	TcdAB	Toxin	↓Pyruvate	[179]
C. difficile	Slec	Germination	↑Taurocholate	[180]
C. difficile	CD2344	Respiration	↑Succinate	[181]
C. difficile	PrdB	Nutrient utilisation	↑Proline	[139]
C. difficile	tcdB/Spo0A	Toxin/sporulation	↓Secondary bile acids DCA LCA	[182]
V. cholerae	tcpA	Pilus/colonisation	↑Autoinducer-2 (AI-2) synthase	[183]
V. cholerae	rtxA/hylA	Toxin	↑Autoinducer-2 (AI-2) synthase	[183]
V. cholerae	flrA	Motility	↑Cholesterol	[184]
V. cholerae	ctxAB/tcpA	Toxin/motility	↓Sodium	[185]

↑Upregulates. ↓Downregulates.

Accumulating evidence over the past decade has linked the high-fat/high-sugar/low-fibre “Western diet” with a myriad of the ever-increasing glycemic index and metabolic disorders. It is widely acknowledged that the microbial dysbiosis resulting from this lifestyle is a major contributing factor to the epidemic of glycemic index and metabolic disorders^[13]^. One could speculate whether long-term dietary regimes could increase or decrease the host’s colonisation resistance to enteric pathogens or opportunistic pathobionts. In this review, the interactions among diet, the microbiota, colonisation resistance and pathogen performance will be examined by focusing on the keystone taxa and metabolites involved.

## BREAST MILK AND SOLID FOODS IN EARLY LIFE PROTECT AGAINST ENTERIC INFECTION BY MODULATING THE GUT MICROBIOTA

Neonates face an increased susceptibility to GI infections. This susceptibility in newborns has been generally attributed to the immaturity of the adaptive and innate immune systems; premature newborns often display a heightened risk of suffering from excessive inflammation, which decreases as they age^[14]^. Given that the gut microbiota is involved in the development of the immune system^[15]^ and the neonatal microbiota is less diverse than the adult microbiota, research is shifting toward the potential role that specific gut taxa play in the maturation of immune function and colonisation resistance.

**Figure 1:**
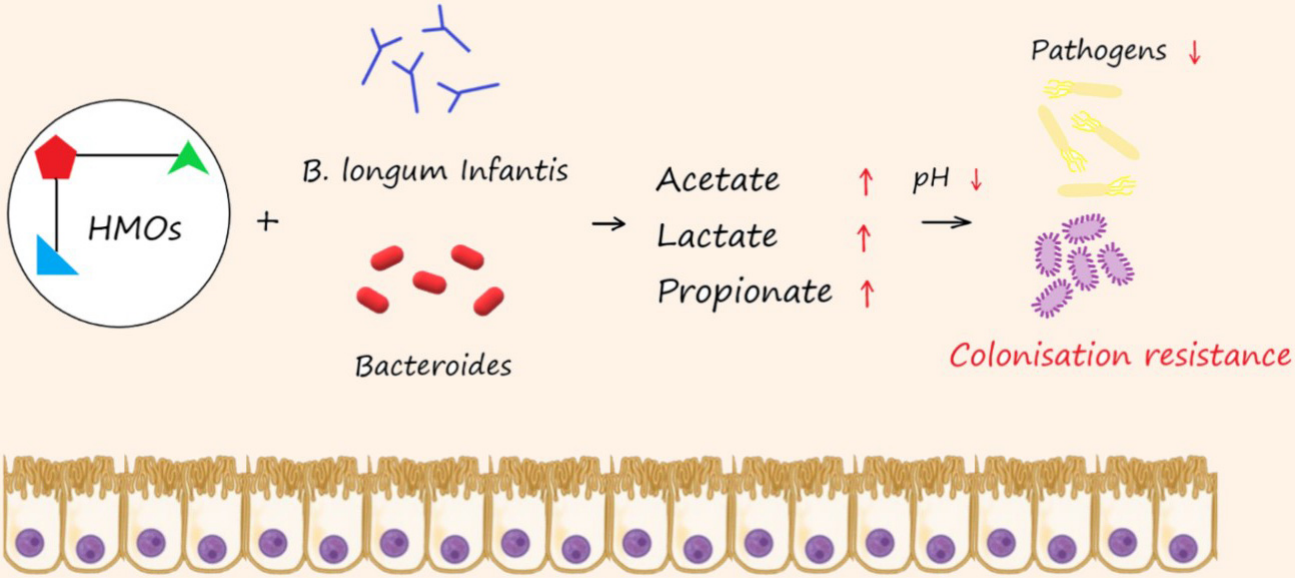
Metabolism of human milk oligosaccharides (HMOs) lowers gut pH boosting colonisation resistance; HMOs are metabolised by initial colonisers (*Bifidobacterium longum *ssp. *infantis *and *Bacteroides*) in the infant gut, producing SCFAs and subsequently lowering pH & increasing colonisation resistance against gastrointestinal pathogens. SCFAs: Short-chain fatty acids.

Naturally delivered infants are generally dominated by bacterial groups associated with maternal vaginal microbiota (e.g., *Atopobium*, *Bacteroides*, *Clostridium*, *E. coli*, *Streptococcus *spp. and *Prevotella*), whereas C-section-born infants are dominated by the taxa associated with the skin microbiota such as *Staphylococcus *spp.^[16]^. Breastfeeding aids in the initial colonisation of key taxa: *Bifidobacterium *and *Lactobacillus*^[17]^, with the former involved in the digestion of human milk oligosaccharides (HMOs), which are resistant to human enzymatic digestion^[18]^. The resulting fermentation of these compounds produces lactate^[19]^ and SCFAs, specifically acetate, which accounts for 80% of the total SCFA production in the infant gut^[20]^ compared with 50% in the adult gut^[21]^. The acidic end products of HMO fermentation are linked to a lower intestinal pH and may be crucial to maintaining colonisation resistance in infants [Figure 1]. The interactions between pathogens and SCFAs will be discussed later.

The distinction between microbiota community composition of human breast milk-fed and formula-fed infants is clear, with the general consensus that human breast milk directs the propagation of beneficial bacteria and their related metabolites^[22]^. Lactation drives the colonisation of *Bifidobacterium *in infants, and has been shown to play a key role in the maturation of the immune system (for a review, see Ref.^[23]^). Moreover, in mice, IgG in breast milk derived from mothers previously infected with *Citrobacter rodentium*, is passed to the offspring, enhancing colonisation resistance when challenged by this pathogen^[24]^. Probiotics derived from breast milk have shown great promise in mitigating the risk of necrotising enterocolitis (NEC), the most common GI disease in preterm infants and the leading cause of death in extremely preterm infants from 2 weeks to 2 months of age^[25]^. Perhaps the most promising “probiotic” is *Bifidobacterium longum *subsp. *infantis*, an extremely proficient coloniser of the infant gut, and exhibits decreasing NEC incidence in neonates^[26]^. More recently, a human breast milk-derived commensal *Propionibacterium* strain UF1, belonging to the same phylum as *Bifidobacterium*, the Actinobacteria, has been shown to mitigate NEC-like injury in mice^[27]^ and conferred protection against *Listeria monocytogenes* infection^[28]^.

Along with *Bifidobacterium*, the other key taxa involved in the fermentation of HMOs are *Bacteroides*^[29]^. Vaginal birth and breastfeeding significantly improve *Bacteroides *colonisation^[30]^, suggesting a long coevolved symbiotic relationship between the host and taxa, and this symbiosis is reflected in studies demonstrating their role in immune system development^[31,32]^. For example, the immunosuppressive effect of the capsular exopolysaccharide, polysaccharide A from *B. fragilis *is achieved by promoting differentiation of regulatory T cells (T_reg_)^[32]^, which may be beneficial to the host given that many pathogens favour a gut-inflamed environment. However, the supposed reliance on *Bacteroides* spp. for the development of the infant immune system is not clear, as an observational study in Northern Europe observed reduced prevalence of *Bacteroides* and Type I diabetes (T1D) in Russian children, relative to their counterparts from Finland and Estonia, where *Bacteroides *and T1D were more common^[33]^. The Bacteroidetes phylum primarily produces acetate and propionate^[34]^. Acetate has been observed to decrease the frequency of autoreactive T cells, and a diet designed to release large amounts of acetate protected against the development of T1D in mice^[35]^. However, *Bacteroides *spp. that may be a risk factor for T1D, might be unrelated to the production of acetate and its associated metabolites, but more likely related to the overexposure to *Bacteroides *- associated LPS derived from HMO-utilising *Bacteroides*^[33]^.

As the diet changes from maternal milk to fibre-rich foods as the infant matures, the infant microbiome acquires members of *Bacteroidales* and the butyrate-producing *Clostridiales*. *Clostridium *species have been associated with the increasing abundance and activity of T_reg_ cells in the colon^[36]^ by providing bacterial antigens. Administration of members of the *Clostridiales*, but not *Bacteroidales*, provided protection to germ-free adult mice, colonised with neonatal microbiota, against infection from *Salmonella *and *Citrobacter*^[37]^. These researchers hypothesise that in the first days of life, oxygen consumption by aerobic bacteria or facultative anaerobes enhances the ability of the strict anaerobes, *Clostridiales*, to colonise the gut, which, in turn, provides protection against pathogen infection. Furthermore, when succinate was administered in the drinking water to mice, it reduced oxygen intestinal content, which in turn, enhanced *Clostridales *colonisation. Interestingly, the authors demonstrated that it was these bacterial groups that abrogated infection and that host immunity did not contribute to the *Clostridia*-mediated effect. Similarly, a longitudinal study examining faecal samples from an asymptomatic infant carrier of *Clostridioides difficile* (an infant female born by C-section), from pre-weaning to weaning, revealed a dramatic change in microbiota composition within the first five days of transition from breast milk to cow’s milk and solid foods^[38]^. A rapid decline and eventual disappearance of *C. difficle*, accompanied by an increase in the relative abundance of *Bacteriodales*/*Clostridiales* observed during weaning, were likely responsible for the expulsion of *C. difficile*.

## THE ROLE OF DIETARY FIBRE IN PROMOTING MICROBIAL DIVERSITY, MAINTAINING THE MUCUS BARRIER AND THE LINK WITH COLONISATION RESISTANCE

There is an appreciation that the diet of westernised nations has declined in the quantity of fermentable fibre intake, which has been associated with the coincidental rise in diseases such as heart disease, diabetes and colorectal cancer^[39]^. Human populations with a diet rich in dietary fibre exhibit increased diversity of the microbiota, such as experienced by the Hadza tribal people from Tanzania^[40]^. Conversely, a “westernised” low fibre diet can result in a progressive loss of microbial diversity^[41]^. While there is a greater prevalence of GI pathogen infections in developing countries compared with their western counterparts^[42]^, this may be attributed to poverty-related risk factors and sanitation practices. Interestingly, in a study comparing the microbiota of children from Burkina Faso and their European counterparts, it was observed that *Enterobacteriaceae *(*Shigella*/*Escherichia*) were underrepresented in the Burkina Faso cohort relative to European children^[43]^. The authors hypothesised that the high fibre diet consumed by the Burkina Faso children selects for a bacterial community capable of maximising energy intake from the fibres, while at the same time protecting them from potential enteropathogens. However, whether diet-induced increases in microbial diversity or specific taxa and their related metabolites can improve colonisation resistance in humans is an intriguing but essential question to answer. The majority of studies to date supporting the inverse link between diversity and infection stem from murine models. Both germ-free and antibiotic-treated mice display increased susceptibility to enteric infections, which are associated with reduced microbiota diversity^[44,45]^. While the bulk of studies supports microbial-induced benefits derived from fermentable fibre, recent evidence suggests that cellulose, an insoluble fibre, also exerts enrichment in protective microbial species and provides colonisation resistance^[46]^. A comparison of the impact of soluble dietary fibre (oat β-glucan) versus insoluble dietary fibre (microcellulose) in mice gut microbiota reported reduced alpha-diversity (distribution of species abundances) and higher relative abundance of fibre-degrading *Bacteroides* and pathogenic Proteobacteria in the former^[47]^, indicating potential cross-feeding between commensals and pathogens. Higher alpha-diversity was observed when the two fibres were mixed, and may be explained by an increase in carbon sources providing substrates for a larger range of bacterial taxa. Such mixtures would reflect better a human diet, which contains a mixture of soluble and insoluble fibres.

SCFAs are the end products of the fermentation of dietary fibres and have a broad range of effects on mammalian host physiology, can attenuate inflammation, and alter the microbial composition and pathogen virulence. SCFAs are saturated aliphatic organic acids comprised of one to six carbons, of which acetate (C2), propionate (C3) and butyrate (C4) are the most abundant (> 95%)^[48]^. The success of the invading pathogen depends on the biotic interactions within the community, including exchanging and competing for metabolites^[49]^. The effect of SCFAs can be a double-edged sword for invading pathogens, with beneficial or inhibitory effects depending on concentration and environmental pH.

The glycoprotein-rich layer that covers the gut epithelium provides the first line of defence against both commensal and pathogenic bacteria. Evidence has suggested that reduced dietary fibre intake is associated with a thinner colonic mucus^[50]^. Indeed, a recent study demonstrated that during chronic or intermittent fibre deficiency in mice, the gut microbiota degraded the host-secreted glycoproteins as an energy source, and in turn, resulted in an unstable mucus barrier function, increasing susceptibility to infection by *C. rodentium*^[51]^. One of the key players in the mucosal-microbiota environment is *Akkermansia muciniphila*, comprising 1%-4% of colonic microbes, which preferentially degrades mucin as its primary nutrient source^[52]^ and is inversely correlated with a myriad of GI diseases. The distribution of *Akkermansia* in the GI tract of vertebrates is vast^[53]^, suggesting a long-term co-evolutionary relationship with their hosts and underlining their symbiotic importance. Mucus consists primarily of the heavily O-glycosylated protein, mucin 2, which *Akkermansia *can degrade with an arsenal of enzymes^[54]^. Continuous production of mucin by the goblet cells contributes to both mucin presence in the mucus layer and in the colonic lumen^[55]^. *Akkermansia* turnover of mucin contributes to the maintenance of intestinal integrity and microbial community homeostasis. Other species, including *Bacteroides thetaiotaomicron *and *Bifidobacterium bifidum*, that possess the capacity to break down mucus O-glycans have been identified in some studies^[56]^. Acetate producers like *B. thetaiotaomicron* may require a commensal adjuvant, e.g., *Faecalibacterium prausnitzii*, an acetate consumer and butyrate producer, in order to maintain colonic epithelial homeostasis^[57]^. *F. prausnitzii *is capable of immunosuppression through blocking of NF-κB activation and anti-inflammatory cytokine production, and reduced abundance of *F. prausnitzii *has been observed in inflammatory bowel disease (IBD) subjects^[58]^, indicating a harmonious relationship between mucosal commensals and the host in a healthy gut environment. Conversely, the glycans released from the mucin may actually provide a food source for GI pathogens. Ng *et al.*^[59]^ demonstrated that *Salmonella *and *C. difficile* thrived on *B. thetaiotaomicron*-liberated glycans following antibiotic-induced disruption of mono-colonised mice compared to germ-free mice.

The western-style diet (high-fat/low-fibre) has been associated with a decrease in *Bacteroides*, *Bifidobacterium *and *Akkermansia*^[60]^, subsequently affecting intestinal mucosal homeostasis and permeability; the effects of which can be ameliorated by the addition of dietary fibres^[60]^, and thereby potentially protecting against infection^[51]^. Moreover, the Western diet is characterised by an increase in the Firmicutes/Bacteroidetes ratio and weight gain in humans. A link between obesity and risk of infection with the enteric pathogen *C. difficile *has been identified^[61]^, and *C. difficile* colonisation has been attenuated in mouse models by the addition of dietary fibre^[62]^, suggesting a fibre deficient lifestyle may be a risk factor for *C. difficile *infection (CDI) and persistence. Collectively, these data suggest that individual fibres select for bacteria that are best at metabolising the specific fibre, leading to reduced diversity and hence a higher risk for colonisation of pathogens. Mixtures of dietary fibres that better represent a human diet promote higher gut microbiota diversity, and thus improve colonisation resistance and mucosal integrity.

## THE DOUBLE-EDGED SWORD OF SHORT-CHAIN FATTY ACIDS

Of the three major SCFAs, acetate is the most abundant, constituting approximately 60% in the colon and stool^[63]^. It can be produced from pyruvate via acetyl-CoA by most of the enteric bacteria (*Akkermansia muciniphila*, *Bacteroides *spp., *Bifidobacterium *spp., *Prevotella* spp., *Ruminococcus *spp.) or from pyruvate via the Wood-Ljungdahl pathway (*Blautia hydrogenotrophica*, *Clostridium *spp., *Streptococcus *spp.)^[21]^. Acetate may have anti-inflammatory effects *in vivo*, by decreasing the LPS-stimulated TNFα response from neutrophils^[64]^, albeit to a lesser extent than butyrate and propionate.

Many pathogens use SCFAs as environmental cues to determine their biogeographical location within the gut and switch on genes accordingly, e.g., virulence factors to colonise the preferential location. For example, *Salmonella typhimurium *preferably colonises the ileum^[65]^, where the typical concentration of acetate is 30 mM. This concentration enhances the expression of SPI-1 (*Salmonella *Pathogenicity Island 1)-encoded T3SS (Type three secretion system), which is involved in the invasion of the host. Similarly, *SPI-1* gene expression is promoted in the presence of minute concentrations of formate (~8 mM), like those encountered in the ileum^[66]^, suggesting *S. typhimurium *has multiple mechanisms to determine its biogeographical location. Furthermore, streptomycin-treated mice were more susceptible to *S. typhimurium *infection in the ileum compared with untreated mice, where the SCFA concentrations remained unchanged, suggesting that ileal commensal bacteria can also affect *S. typhimurium *virulence, likely by physically blocking colonisation or contributing to the immune response^[67]^. On the contrary, higher concentrations of propionate and butyrate, or the absence of formate, i.e., similar to conditions found in the colon, suppress the expression of T3SS^[68]^, and invasion is inhibited. Colonic environmental cues likely initiate adaptation of *S. typhimurium* gene expression to endure environmental insults and/or preparation for transmission to a new host. Interestingly, a recent study observed reduced ileal colonisation of *Salmonella* in mice which were pre-treated with a consortium of *Bacteroides* spp. with a high capacity for production of propionate^[69]^, through disruption of intracellular pH homeostasis.

Similarly, Enterohaemorrhagic *E. coli* (EHEC) utilises SCFAs for virulence gene regulation; its preferred site of colonisation and infection is the colon^[70]^, where the ratio of acetate/butyrate tends to be lower. However, some studies have demonstrated that acetate can be refractory to the virulence of EHEC^[71]^ by lowering intestinal pH^[72]^. Mixtures of SCFAs that represent the small intestine significantly upregulate EHEC flagellar genes and motility, whereas colonic SCFA concentrations have a down-regulatory effect^[73]^. Expression of the *iha* gene that encodes an adherence-conferring outer membrane protein, however, is upregulated by EHEC in the small intestine^[74]^, and is crucial for colonisation and infection. Consequently, ileal SCFA concentrations activate EHEC flagellar production and motility, followed by expression of genes involved in type III secretion and adherence when approaching colonic SCFA concentrations^[75]^, thereby permitting efficient adherence in EHEC’s preferred niche. Production of acetate by *Bifidobacterium *has been demonstrated to inhibit the translocation of Shiga toxin of the EHEC 0157:H7 from the gut lumen^[76]^ and prevent 0157:H7-induced colonic epithelial cell death via *Bifidobacterium *acetate-upregulated carbohydrate transporters^[77]^. *Campylobacter jejuni *has similar mechanisms to sense metabolites and hence spatial distribution. In avian hosts, where *C. jejuni* behave as symbionts in the lower GI tract, concentrations of acetate are high and allow for the expression of genes that permit commensal colonisation^[78]^. Conversely, high concentrations of lactate, similar to those observed in the upper GI tract, where *C. jejuni *colonises less efficiently, repress the genes involved in colonisation. The authors speculate whether *C. jejuni *utilises similar environmental cues in order to colonise humans and, thereby, cause diarrhoeal disease.

SCFAs have also been associated with concentration-dependent negative effects on *C. difficile *growth^[79]^, and as SCFA concentrations are reduced following antibiotic treatment, this could be a contributing factor to its subsequent colonisation. Other studies have demonstrated that SCFAs increase the expression of Toxin B (TcdB), an essential virulence factor^[80]^. SCFAs may serve as a signal to *C. difficile *of an inhospitable and competitive environment; therefore, upregulation of TcdB may provide a survival mechanism. The success of faecal microbiota transplants (FMT) in the treatment of CDI reinforces the role of commensal microbiota (*Bacteroides*, *Clostridium *clusters IX and XIVa) in the treatment of CDI^[81]^. Early *in vitro *studies have demonstrated that dietary fibre polysaccharides induce a bifidogenic effect and hence increase SCFA production, which may result in enhanced colonisation resistance against *C. difficile*^[82]^. Likewise, an *in vivo *study found that *C. difficile*-infected mice fed a diet rich in dietary fibre had stimulated the growth of fibre-utilising taxa (*Bacteroides *spp.) and their associated metabolites, i.e., SCFAs^[62]^, with decreased *C. difficile *fitness and numbers, while toxin expression was increased^[62]^.

Among the major SCFAs, butyrate is the most extensively studied, largely due to its beneficial effects on both colonocyte energy metabolism and intestinal homeostasis^[83]^. Butyrate is the least abundant of the three SCFAs produced, comprising 15% of the total SCFA pool in humans^[84]^. Butyrate can upregulate mucin 2, reinforcing the mucus layer of the intestinal mucosa, and leading to enhanced protection against luminal pathogens^[85]^. Butyrate is formed in the so-called “classical pathway”, by the condensation of two molecules of acetyl-CoA, and the subsequent reduction to butyryl-CoA, which can be converted to butyrate by members of the *Clostridia *family (*Anaerostipes* spp., *Coprococcus catus*, *Eubacterium rectale*, *Eubacterium hallii*, *F. prausnitzii*, *Roseburia *spp.)^[21,86]^. *Anaerostipes* spp. and *E. hallii *are also capable of utilising lactate as the substrate for the production of butyrate. Alternatively, butyrate can be synthesised from butyryl-CoA by the phosphotransbutyrylase/butyrate kinase route (*Coprococcus comes*, *Coprococcus eutactus*)^[86]^.

Butyrate is at its highest concentration in the colon, and specific pathogens use the high concentrations of butyrate as an environmental cue in order to express virulence factors. For example, EHEC exhibited high adherence to Caco-2 cells in the presence of butyrate, whereas acetate and propionate had little effect^[87]^. Similarly, Shiga toxin-producing *E. coli* (STEC) exhibit increased adherence in the presence of SCFA concentrations that reflect those that are found in the colon^[74]^. In a study by Zumbrun *et al.*^[88]^, the authors demonstrated that a high fibre diet contributed to elevated butyrate and reduced commensal *Escherichia* compared to a low fibre diet; these resulting changes led to higher STEC colonisation, more weight loss and higher mortality in high fibre diet-fed mice. In IBD patients, faecal butyrate concentrations are higher than healthy controls, despite the lower abundance of butyrate-producing taxa^[89]^, and this may be explained by its impaired uptake and oxidation by inflamed colonocytes. While colonic SCFA concentrations seem to exacerbate certain *Escherichia *pathologies, colonic concentrations of butyrate^[90]^ and propionate^[91]^ have an antagonistic effect towards invasion gene expression in *Salmonella*, by down-regulating expression of SPI-1.

The butyrate-producing *Clostridia *are obligate anaerobes capable of maintaining healthy gut homeostasis. Under eubiosis, the *Clostridia*-derived butyrate is the major energy source for the colonocytes and activates epithelial signalling through the intracellular butyrate sensor PPAR-y, driving mitochondrial β-oxidation of this substrate. *Salmonella *virulence factors induce inflammation during the early stages of infection, and these virulence factors have been shown to deplete the butyrate-producing *Clostridia *from the gut-associated community, leading to an epithelial aerobic environment which ultimately favours the aerobic expansion of the pathogen^[92]^. Moreover, antibiotic treatment resulting in a reduction of PPAR-y signalling, i.e., increased bioavailability of oxygen, has been shown to exacerbate this effect^[93]^. The reduced butyrate concentrations observed during *Salmonella *infection stimulate the colonocytes to switch from β-oxidation of butyrate to lactate fermentation and increase luminal lactate. *Salmonella *exploits this increase in lactate and utilises this carbon source for subsequent expansion^[94]^. Recently, genes involved in the direct β-oxidation of butyrate have been identified in *Salmonella*, and excision of the operon drove the transition from a GI to an extraintestinal pathogen, i.e., non-typhoidal to typhoidal^[95]^, suggesting utilisation of butyrate plays a crucial role in *Salmonella* GI disease. Moreover, specific members of *Clostridia *are some of the few bacterial species capable of utilising fructose-asparagine, a known food source for *Salmonella*, which improves its fitness^[96]^, and could explain an evolutionary competition between these species.

In silico analysis of butyrate production pathways in GI pathogens has identified members of the *Fusobacterium* genus and a few pathogenic strains of *Clostridium* (*C. tetani *and *C. tetanomorphum*) with the ability to produce butyrate^[97]^. However, their capacity to synthesise butyrate involves amino acids, primarily glutamate and lysine, as substrates, and are different to those observed in commensals, which primarily ferment pyruvate for butyrogenesis. The end product of this amino acid fermentation yields ammonia, higher concentrations of which are associated with colorectal cancer (CRC)^[98]^. Additionally, increased *Fusobacterium nucleatum *abundances have been observed in CRC patients when compared with healthy controls, and has been suggested as a possible microbial biomarker in CRC development^[99]^. It remains to be seen whether CRC tumorigenesis is a cause or consequence of microbiota alterations; however, there are associations of an “inflammatory diet”, i.e., high consumption of red meat, processed meat and refined grains, with the prevalence of *F. nucleatum*-positive colorectal carcinomas^[100]^.

**Figure 2:**
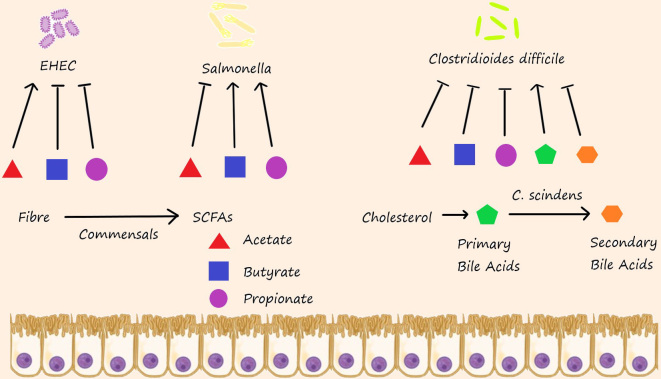
Differential effects of dietary metabolites on pathogens; metabolites from fibre degradation by commensals have differential effects on the success of an invading pathogen. Acetate promotes the growth of Enterohemorrhagic *E. coli *(EHEC), whereas butyrate and propionate repress growth. Conversely, acetate represses *Salmonella* growth, whereas butyrate and propionate promote growth. Colonisation of *Clostridioides difficile *is increased by the cholesterol metabolites, primary bile acids. The commensal *Clostridium scindens *can limit the availability of primary bile acids by converting these to secondary bile acids, thus increasing colonisation resistance to *C. difficile*. SCFAs: Short-chain fatty acids.

On the whole, the effects of SCFAs on pathogen colonisation are concentration-dependent, with higher or lower concentrations having the capacity to be either antagonistic or hospitable, respectively, depending on the species and its preferred niche. Some examples of these differential effects of SCFAs can be found in Figure 2. The studies mentioned here only skim the surface on the complexity of the microbial and chemical interactions in the microbiota that influence health and disease.

## PREBIOTICS

Prebiotics are selectively fermented ingredients that beneficially affect the host by stimulating the growth and/or functional activity of one or a limited number of bacteria in the colon, and thus improve host health^[101]^. The premise that these can potentially inhibit or obstruct the growth or virulence of pathogens is nothing new, and has been suggested as an alternative to antibiotic growth promoters in animals. The best-documented benefits stem from the use of indigestible oligosaccharides, such as fructans and galactans^[102]^; however, consideration into the ability of pathogens to ferment or utilise the prebiotics or the associated metabolites should be taken into account^[103]^. Cereals, fruits and legumes are natural sources of prebiotics, whilst the active components are often synthesised using industrial chemical and enzymatic methods. The majority of prebiotic studies on pathogen inhibition to date involve livestock and animal models, which vary in terms of physiology and microbiota composition; therefore, care should be taken in translating these results to humans.

Prebiotics such as fructo-oligosaccharides (FOS) and galacto-oligosaccharides (GOS) are preferentially fermented into SCFAs by *Bifidobacterium *and *Lactobacillus* which have historically been viewed as beneficial bacteria, resulting in lowered luminal pH. The addition of oligosaccharides to poultry feed has been shown to increase *Bifidobacterium *and *Lactobacillus* populations, while also being refractory to *E. coli*^[104]^ and *Salmonella* colonisation^[105,106]^. This outgrowth of prebiotic-utilising taxa has also been demonstrated to eliminate *C. difficile* in mice^[62]^. The prebiotic inulin is primarily composed of FOS and has been shown to ameliorate low-grade inflammation through microbiota-dependent induction of IL-22 expression^[107]^ and was able to prevent increased bacterial mucus penetration in high-fat diet-fed mice^[108]^. Furthermore, supplementation of high-fat diet-fed mice with *B. longum *restored mucus growth^[108]^, suggesting that prebiotic and probiotic treatments have the potential to prevent intestinal mucus abnormalities, which is a consequence of a high-fat diet. However, a study by Miles *et al.*^[109]^ demonstrated that inulin may actually have the potential to exacerbate disease severity in response to inducers of colitis such as dextran sodium sulphate (DSS) in both low-fat and high-fat diets. Moreover, inflammation is crucial for the successful colonisation of *Salmonella*, through the inflammatory-mediated expression of virulence factors; studies in rats have shown increased translocation of *Salmonella *when FOS is added to the diet^[110]^. Although the overwhelming data suggest the beneficial effects of inulin supplementation, more care is needed to define the specific mechanisms by which inulin impact the gut microbiota to protect against the effects of inflammation and improve mucosal integrity.

The other extensively studied prebiotic is GOS, which is commercially produced from lactose using glycoside hydrolases that catalyse transgalactosylation reactions^[111]^. *In vitro *studies have demonstrated the protective effect of GOS against EHEC and *Cronobacter sakazakii*, through an anti-adherence mechanism^[112]^. Interestingly, a recent study on the *in vivo *protective effects of GOS on the murine EHEC model pathogen, *C. rodentium*, showed that GOS treatment prevented pathogen-induced intestinal tissue damage, independent of anti-adherence activity and *C. rodentium *abundance^[113]^. In terms of the effect of GOS on microbiota composition, studies have identified a clear bifidogenic effect of GOS, while simultaneously lowering *E. coli*, *Helicobacter *and *Clostridium *spp. abundances^[114]^. The combined effects of the prebiotic on SCFA production, microbiota composition, pathogen virulence and fitness make it difficult to pinpoint the exact mechanism involved in providing a protective effect. This lack of a known mechanism underscores the importance of thorough analysis in animal studies before extrapolating results to humans.

## DIETARY LIPIDS AND BILE ACIDS

Bile acids are amphipathic biological detergents produced by the liver with the primary function of metabolising lipids in the GI tract^[115]^, and their production is linked to the ingestion of fatty foods. Those bile acids, which are not reabsorbed into the liver (~5%), can serve as substrates for colonic microbial metabolism, i.e., hydrolysis of conjugated bile acids by bile salt hydrolases or biotransformed into secondary bile acids by 7α-dehydroxylation, where they are either excreted in faeces or recirculated back into the liver through the enterohepatic circulation^[116]^. Thus, changes in microbiota composition culminate in changes in the bile acid pool, and this homeostatic imbalance is associated with a range of disease states, including CRC, IBD and recurrent CDI^[117]^. Recent advances have identified specific operational taxonomic units, i.e., closely related bacteria, involved in bile acid biotransformations and correlate to a loss of specific taxa with the development of disease. For example, the previous infection of mice with *Yersinia pseudotuberculosis *remodels the microbiota to enrich for Deltaproteobacteria, a taurine metabolising class of bacteria which provide colonisation resistance to the pathogen *Klebsiella pneumoniae*^[118]^*.* Thus, commensal bile acid interactions are intrinsically linked in both mitigation and amplification of colonisation resistance. Identifying the biochemical mechanisms which underpin the effect on colonisation resistance will improve our knowledge going forward and open up new avenues for therapeutic manipulation of the microbiota.

Antibiotic-induced destruction of the microbiota is associated with recurrent CDI. The protective role of the microbiota against *C. difficile *can be consolidated by the success of FMT^[81]^. *C. difficile *spores must germinate *in vivo *to develop into actively growing bacteria to produce enough toxins to initiate infection. *In vitro*, primary bile acids stimulate germination, and secondary bile acids inhibit this process^[119]^. Indeed, these interactions [Figure 2] have been shown *in vivo*, whereby depletion of secondary bile acids in the ileum resulted in *C. difficile *germination and growth^[120]^. Moreover, inflammation induced by *C. difficile *toxins subsequently changes this pathogen’s nutrient metabolism pathways and enables it to thrive in the inflamed gut, particularly on products of collagen degradation, outcompeting commensal bacteria aside from members of the *Bacteroides *genus, which can also utilise collagen degradation products^[121]^. In a study by Buffie *et al.*^[122]^, the authors identified a single commensal that conferred resistance to *C. difficile*; *Clostridium scindens*, a bile acid 7α-dehydroxylating intestinal bacterium, which enhanced resistance in a secondary bile acid-dependent fashion. *C. scindens*-mediated restoration of secondary bile acids from host-derived bile salts were sufficient in inhibiting *C. difficile *germination, underpinning the pivotal role commensal bile acid-metabolising bacteria play in preventing recurrent CDI. Patients successfully treated for recurrent CDI have an enrichment of bile salt hydrolase-producing bacteria, the abundance of which negatively correlates with faecal concentrations of taurocholic acid, a primary bile acid^[123]^. While obesity has been identified as a risk factor for CDI^[61]^, and the microbiota composition of obese subjects is characterised by a decrease in the Bacteroidetes/Firmicutes ratio, there is no evidence to suggest that bile acid synthesis or enterohepatic circulation is altered by obesity. However, an obesity-driven altered and less diverse microbiota coupled with antibiotic elimination of bile-acid metabolising bacteria may very well contribute to an increased risk of CDI.

High-fat diets promote the biosynthesis of bile, which can impact commensal microbiota that are sensitive to bile acid concentrations. Additionally, pathogens such as *S. typhimurium *are quite resilient to high bile acid concentrations, and colonisation resistance to this pathogen is alleviated upon oleic acid or high-fat diet supplementation in mice^[124]^, and colonisation resistance is improved when switched back to a plant-based diet. Commensal *E. coli *that can compete with *S. typhimurium *through bile acid^[124]^ or oxygen^[125]^ competition could provide a means of protection against this pathogen.

## PROTEIN AND AMINO ACIDS

Aberrations in microbiota community structure driven by antibiotics, infection and/or diet will affect protein homeostasis and can increase free amino acids in the gut, providing a nutritional niche upon which some pathogens can capitalise. Indeed, many gut pathogens such as EHEC^[126]^, *Vibrio cholerae*^[127]^, *C. jejuni*^[128]^, and *C. rodentium*^[129]^ have genes involved in amino acid biosynthesis upregulated upon gut colonisation. Moreover, the host relies on amino acid metabolism to support its immune responses against invading pathogens, with diets deficient in protein having a counterproductive effect on immune function, independent of the microbiota^[130]^. On the other hand, dietary administration of high protein and amino acid by-products stimulates the growth of pathogens^[129]^ and protein-fermenting bacteria contribute to disease susceptibility^[131]^. Therefore, identifying commensal competitors and pathogen metabolic pathways involved in protein fermentation and amino acid biosynthesis may help in developing new strategies to encourage colonisation resistance through diet.

D-amino acids are biosynthesised by gut bacteria as opposed to L-amino acids which humans biosynthesise or obtain from the diet. Tryptophan is an essential amino acid from the diet in mammals and is primarily catabolised by commensal bacteria into various indole-containing metabolites. While tryptophan is required for optimal immune responses, such as T-cell proliferation, the commensal-mediated tryptophan metabolites can have differential effects on gut pathogens. In *S. typhimurium*, the tryptophan metabolite indole induces expression of genes related to efflux-mediated multidrug resistance^[132]^, while concomitantly decreasing the expression of genes involved in invasion located on the SPI-1 pathogenicity island^[133]^. On the other hand, indole upregulates EHEC secretion of EspA and EspB via the type III secretion system, enhancing this pathogen’s ability to form attaching and effacing (A/E) lesions^[134]^, while other indole-derivatives can inhibit biofilm formation, motility and formation of A/E lesions^[135]^. The enzyme indoleamine 2,3-dioxygenase (IDO) catalyses the conversion of tryptophan into kynurenine, reducing the tryptophan pool in the gut, and thereby directly impacting various immune responses. *C. difficile *infection upregulates the expression of IDO, increasing the production of kynurenine, subsequently depleting the tryptophan pool, and thereby diminishing the immune responses of the host toward this pathogen^[136]^. All in all, tryptophan and its associated metabolites have direct effects on immune function and pleiotropic effects on gut pathogens, suggesting that this molecule will be of interest in colonisation resistance studies moving forward.

Many gut pathogens switch metabolic pathways depending on the environment, e.g., in inflammation. The pathobiont Adherent Invasive *E. coli *(AIEC) shifts its metabolism to catabolise L-serine in the inflamed gut to maximise growth potential^[137]^, with L-serine having little effect on AIEC fitness in a healthy gut environment. Interestingly, AIEC bloom in the inflamed gut, and are significantly reduced when amino acids are decreased in the diet. *C. difficile *exploits the niche created following particular antibiotic treatments, and this dysbiotic environment has increased the availability of amino acids. Specifically, a recent study demonstrated that *C. difficile *is dependent on L-proline metabolism, as L-proline knockout *C. difficile *strains were unable to colonise the gut of germ-free mice transplanted with either a dysbiotic or healthy microbiota^[138]^. Furthermore, low-protein or low-proline diets given to mice substantially decreased wild-type *C. difficile *expansion suggesting that *C. difficile *is dependent on proline for adequate colonisation, which can potentially be mediated through dietary intervention. Furthermore, commensals such as members of the *Clostridia *class, decreased the fitness advantage of *C. difficile*’s ability to ferment proline, through competition for this amino acid^[139]^. Likewise, EHEC was found to be reliant on proline for colonisation, with commensal *E. coli *that compete for proline attenuating the expansion of EHEC^[140]^.

As discussed above, the microbiota can limit the colonisation of invading pathogens by depleting the concentration of amino acids in the gut. Some pathogens can overcome this problem by inducing amino acid biosynthesis to subvert such a deficiency. Transposon sequencing is a powerful tool and allows for the generation of a library of random pathogen mutants, for example, those defective in amino acid biosynthesis pathways^[141]^. This technique provides a means to estimate the fitness contribution or essentiality of each genetic component in a bacterial genome. Caballero-Flores *et al.*^[129]^ applied this to *C. rodentium* and found that specific mutants deficient in the production of arginine, threonine, histidine, tryptophan, or isoleucine lost their competitive advantage in mice, compared to wild-type *C. rodentium*. Moreover, these genes were significantly upregulated in conventional mice as opposed to germ-free mice, suggesting that *C. rodentium *specifically use these pathways to outcompete the microbiota. Feeding of a high-protein diet to mice produced markedly better colonisation of *C. rodentium *compared to normal chow. While mouse studies like these inform a mechanistic understanding of pathogen colonisation, the importance of these findings in relation to human disease warrant further investigation.

## TRACE ELEMENTS

As mentioned earlier, the majority of human enteric pathogens belong to the phylum Proteobacteria. In the normal intestine, which is largely inhabited by commensals, mainly Bacteroidetes and Firmicutes, Proteobacteria only constitute < 1% of microbiota populations. The outgrowth of Proteobacteria “blooms” are a hallmark of gut “dysbiosis” resulting from microbial perturbations caused by antibiotic therapy, dietary changes or inflammation.

The availability of micronutrient trace elements is essential to the successful colonisation of pathogens during infection. Nearly 60% of known enzymes contain at least one metal cofactor, with zinc being the most common, followed by iron and manganese^[142]^. In the inflamed gut, these dietary trace elements are heavily sequestered by high affinity binding proteins or kept in organelles that are not accessible to bacteria, in a process known as “nutritional immunity”^[143]^. Many proteobacterial pathogens are equipped with an array of high affinity siderophores, to help them overcome the restriction of available metals and ultimately drive key cellular processes, which in turn sustains and propagates infection. Deficiency or increased supplementation of dietary trace elements may disrupt the commensal microbial populations and predispose individuals to infection.

Zinc deficiency is associated with increased *Enterobacteriaceae *and *Enterococcus*, with concomitant decreases in abundance of *Clostridiales* and Verrucomicrobia (*A. muciniphilia*)^[144]^. Moreover, in a mouse model of enteroaggregative *E. coli *(EAEC), a cause of traveller’s diarrhoea, zinc-deficient mice exhibited altered immune responses and an increase in EAEC virulence factors^[145]^. Furthermore, dietary zinc supplementation abrogated disease progression, reduced EAEC colonisation and expression of virulence factors^[146]^. In another study, zinc supplementation protected from uropathogenic *E. coli* haemolysin-induced gut barrier dysfunction^[147]^. These observations indicate a beneficial impact of zinc supplementation on zinc-deficient subjects; conversely, excessive zinc supplementation can have a detrimental impact on microbial homeostasis and host immune responses. In a study by Zackular *et al.*^[148]^ zinc supplementation stimulated the growth of *Enterococcus *and *Clostridium *XI cluster while concomitant reductions in *Turicibacter *and *Clostridium *(unclassified) were observed. Ultimately, excess zinc selected for a microbiota that was much more prone to destruction by antibiotics, thus exacerbating *C. difficile* colonisation and associated disease^[148]^.

For many bacterial pathogens, the availability of iron is often the limiting factor for colonisation and infection. During inflammation, nutritional immunity limits the bioavailability of iron in the gut, and thus bacterial species equipped with an array of iron acquisition systems are often the most successful and pathogenic. Given the ability of bacterial siderophores to hijack host iron homeostasis, it is not surprising that the innate immune system has evolved mechanisms to counteract bacterial iron acquisition, such as the production of Lipocalin-2 (LCN2). In the acute phase response to infection, LCN2 is expressed to bind bacterial siderophores and neutralises bacterial capacity to sequester iron. However, some bacteria have evolved resistance mechanisms to counteract this immune response, such as the stealth siderophore salmochelin produced by *Salmonella*, thereby gaining a competitive advantage in the inflammatory milieu^[149]^. Interestingly, the probiotic *E. coli *strain Nissle shares many fitness properties to uropathogenic *E. coli*, including iron uptake systems. In the presence of LCN2, Nissle is capable of outcompeting *Salmonella* in a mouse model^[150]^, underscoring the evolved synergy between commensal and host immune response in thwarting pathogen colonisation. Some pathogens, such as *V. cholerae*, have the ability to obtain iron from haem only when Cholera toxin (CTX) is produced^[151]^. The production of CTX induces inflammation and thus decreases gut iron concentrations but enables the bioavailability of host haem, while concurrently changing the transcriptomic gene signature of *V. cholerae *to one that is capable of utilising iron from haem. This change allows the expansion of *V. cholerae *by providing an iron-limited metabolic niche and a competitive advantage over commensals by this pathogen’s unique ability to acquire iron from haem.

In both developing and developed nations, iron deficiency remains the most common form of nutritional deficiency, in many cases prompting iron supplementation to alleviate symptoms of malnutrition. Given the importance of iron to GI pathogens, the effect of iron in bolstering colonisation resistance should perhaps be considered as a detrimental effect by inducing microbial dysbiosis. Indeed, an outgrowth of *Enterobacteriaceae *and increased risk of infection has been observed in both mice^[152]^ and humans^[153]^, following iron supplementation. Intriguingly, the adverse effects can be mitigated simply by the addition of prebiotics to the diet^[154,155]^, inducing growth of beneficial *Bifidobacterium *and *Lactobacillus*. *Bifidobacterium* have also been demonstrated to efficiently sequester iron^[156]^. The reliance of the host immune system on sequestration of iron, coupled with commensal sequestration capacity and subsequent SCFA production, play multifactorial roles in reducing pathogen colonisation.

In addition to the production of LCN2, the host can produce another antimicrobial molecule, Calprotectin, whose primary function is to bind to free zinc and manganese in the gut lumen. Bacteria utilise manganese as a cofactor for a number of proteins; perhaps the best studied is the role of manganese as a detoxifier of reactive oxygen species, of which numerous are encountered following an immune response. *Salmonella *has evolved high-affinity cation transporters to bypass the action of calprotectin and therefore promote growth in an inflamed intestine^[157]^. It is unclear how excess or deficient dietary manganese influences gut microbiota populations. It is possible many pathogens behave similarly to *Salmonella *in subverting calprotectin when manganese is in excess, or perhaps behave similarly to *Staphylococcus aureus*, which can switch to iron in manganese-deplete conditions; thereby bypassing nutritional immunity and causing infection^[158]^.

Pinpointing the delicate balance between trace element toxicity and deficiency while simultaneously understanding the mechanisms involved in both nutritional immunity and colonisation resistance remains complicated. Understanding the complex pathways dietary trace elements play in microbial respiration in infection and inflammation will undoubtedly uncover novel treatments. For example, recently, dysbiotic *Enterobacteriaceae *blooms were ameliorated by tungstate treatment, which inhibited molybdenum-cofactor-dependent respiratory pathways and reduced the severity of inflammation in mouse models^[159]^.

## CONCLUSION

Disentangling the direct and indirect impact of dietary ingredients and nutrients on commensal bacteria in the gut, their associated metabolites, immune function and pathogen virulence will no doubt be challenging. This knowledge will require multidisciplinary collaborations between experts in nutrition, immunology, and microbiology, to name a few. Given that gnotobiotic and antibiotic-treated mice are more susceptible to infection, and that this phenotype can be reversed upon supplementation with even a simplified consortium of commensal bacteria^[160]^ strongly supports the paradigm of colonisation resistance. Reductionist or modular approaches like these can help identify potential probiotic or synbiotic candidates and generate insights into diet-microbe/host-microbe/microbe-microbe interactions.

Studies in neonates and infants support the importance of breast milk-feeding, in providing antibodies and colonisation of human breast milk-metabolising taxa, both of which have been shown to enhance colonisation resistance in the offspring. Moreover, C-section delivery disrupts the mother to infant transmission of specific commensals, resulting in a reduced immunostimulatory potential passed to the offspring^[161]^. Perhaps a critical, yet, unexplored area of research would be the impact of infant formula-metabolising taxa in reducing/providing protection against pathogens. Identifying specific species and their associated functions, which improve neonatal colonisation resistance, has the potential to optimise the production of infant formula through the selection of desired carbon sources.

The mucus layer provides the first line of defence against exogenous microorganisms, the integrity of which is greatly determined by microbial composition, which in turn, is influenced by dietary components, in particular fibre. Patients with IBD should be cautious when consuming specific dietary fibre or prebiotics; inulin has been demonstrated to exacerbate DSS-induced colitis in mice, whereas others such as psyllium have been successful in ameliorating gut inflammation^[162]^. In the context of human GI disease, it is equally ambiguous, as a study observed avoidance of dietary fibre associated with flares in Crohn’s disease patients but not in ulcerative colitis patients^[163]^. Research focusing on individual fibres or prebiotics must be interpreted cautiously as they may inadvertently select a small subset of taxa, while care must be taken when comparing mouse chow diet controls, which contain a mixture of fibres, and thus selecting for a larger subset of taxa. More research is required in humans, in terms of the impact of dietary fibre and prebiotics on the function, stability and characterisation of specific taxa and their associated metabolites. These data could complement *in vitro*, *ex vivo* and “humanised” mouse studies to identify mechanisms when the host is challenged with a GI pathogen.

Microbiota alterations, driven either by pathogen-induced inflammation or other diseases such as IBD, will ultimately impair normal SCFA homeostasis through changes in commensal SCFA producers and/or pathogen/pathobiont utilisation of SCFAs. Given that SCFAs provide an energy source for colonocytes and the majority of human studies measure SCFA concentrations from excreted faeces, faecal SCFA concentrations may not be representative of those found in other parts of the GI tract. Thus, extrapolating findings from human dietary intervention studies in providing SCFA-mediated effects should be approached with caution.

Mechanisms of protein or trace element homeostasis in the gut, with respect to interactions with complex and diverse commensal and pathogenic bacteria, are largely uncharacterised, especially in the context of human disease. This uncertainty is further complicated by interindividual variations underpinned by yet-to-be-determined genetic, environmental or epigenetic factors. While simplified mouse models may fail in recapturing the complexities observed in humans, they can be valuable assets in identifying commensal bacteria with, for example, a high degree of affinity for trace elements, e.g., iron siderophores. Uncovering dietary ingredients that promote their growth could, in theory, boost colonisation resistance through nutrient competition. Future research into the role of branched-chain fatty acids derived from the catabolic products of branched-chain amino acids and how they impact colonisation resistance could be an attractive area of research, given that they may influence metabolic homeostasis in the gut.

Seasonal variations in the mouse chow diet, industrial variations in the processing of prebiotics and feed supplements, choice of laboratory pathogen/commensal strains and breed of the mouse all contribute to disparities among research groups. Thus, tightly controlled models are a necessity, before translating to novel therapeutics and functional foods. Moreover, the fine line between commensal and pathogen in genetically predisposed individuals only adds to the uncertainty and personalised dietary interventions^[164]^ in these individuals for the prevention of infection is an interesting prospect, but further research is warranted.

## DECLARATIONS

### Copyright

© The Author(s) 2022.
